# Symmetry-Engineering-Induced In-Plane Polarization Enhancement in Ta_2_NiS_5_/CrOCl van der Waals Heterostructure

**DOI:** 10.3390/nano13233050

**Published:** 2023-11-29

**Authors:** Yue Su, Peng Chen, Xiangrui Xu, Yufeng Zhang, Weiwei Cai, Gang Peng, Xueao Zhang, Chuyun Deng

**Affiliations:** 1 College of Physical Science and Technology, Xiamen University, Xiamen 361005, China; 2 College of Science, National University of Defense Technology, Changsha 410073, China; 3 Songshan Lake Materials Laboratory, Dongguan 523808, China

**Keywords:** Ta_2_NiS_5_/CrOCl, symmetry engineering, van der Waals heterostructure, enhanced anisotropy, angle-dependent Raman spectrum, electrical transport

## Abstract

Van der Waals (vdW) interfaces can be formed via layer stacking regardless of the lattice constant or symmetry of the individual building blocks. Herein, we constructed a vdW interface of layered Ta_2_NiS_5_ and CrOCl, which exhibited remarkably enhanced in-plane anisotropy via polarized Raman spectroscopy and electrical transport measurements. Compared with pristine Ta_2_NiS_5_, the anisotropy ratio of the Raman intensities for the B_2g_, ^2^A_g_, and ^3^A_g_ modes increased in the heterostructure. More importantly, the anisotropy ratios of conductivity and mobility in the heterostructure increased by one order of magnitude. Specifically speaking, the conductivity ratio changed from ~2.1 (Ta_2_NiS_5_) to ~15 (Ta_2_NiS_5_/CrOCl), while the mobility ratio changed from ~2.7 (Ta_2_NiS_5_) to ~32 (Ta_2_NiS_5_/CrOCl). Such prominent enhancement may be attributed to the symmetry reduction caused by lattice mismatch at the heterostructure interface and the introduction of strain into the Ta_2_NiS_5_. Our research provides a new perspective for enhancing artificial anisotropy physics and offers feasible guidance for future functionalized electronic devices.

## 1. Introduction

Low-symmetry two-dimensional (2D) materials exhibit significant anisotropy in optical, electrical, and thermal properties due to their asymmetric lattice structures, which has attracted widespread attention in the past decade [1,2,3,4]. Phosphorus (BP), one of the most famous low-symmetry materials, has been widely used in polarization optoelectronics, sensing, and energy storage [5,6,7]. Moreover, symmetry engineering and artificial anisotropy offer a new degree of freedom to modulate the original physical properties of 2D materials toward improved functional performance. Recently, a study reported a novel symmetry reduction method that employs van der Waals (vdW) interfaces to achieve artificial anisotropy enhancement in ReS_2_ [8]. In this sense, vdW heterostructures can offer a simple and effective approach to reduce the symmetry of 2D materials.

VdW heterostructure interfaces serve as a platform for studying exotic physical properties, which can be easily prepared via the combination and stacking process of diverse layered materials [9]. When lattice mismatch in a heterostructure occurs at a specific angle, moiré patterns can be observed at the interface, which triggers novel physical phenomena that are absent in the parent materials [10,11]. For instance, in a WSe_2_/BP heterostructure, WSe_2_ and BP form periodic moiré patterns via vdW forces, resulting in the in-plane polarization of isotropic WSe_2_. In addition, lattice mismatch at the heterostructure interface introduces strain within the material. By applying uniaxial tensile strain, the structural symmetry of MoS_2_ can be altered, enabling it to successfully exhibit anisotropic characteristics [12]. However, introducing in-plane polarization in highly symmetrical materials results in a lower anisotropy ratio. Therefore, we enhance the anisotropic differences in low-symmetry materials via symmetry engineering.

Ta_2_NiS_5_, a low-symmetry ternary transition metal chalcogenide, has attracted widespread attention due to its applications in electronics, optoelectronics, and biosensing [13,14,15,16,17]. Unlike single-element or binary anisotropic materials such as BP, PtSe_2_, and WTe_2_, ternary chalcogenides consist of three elements and can adjust their physical properties via stoichiometric variation [18,19,20]. However, the anisotropy ratio of Ta_2_NiS_5_ makes it difficult to reach the requirements of practical applications. Therefore, we chose to further enhance the anisotropy of Ta_2_NiS_5_ via symmetry engineering. CrOCl is a low-symmetry antiferromagnetic insulator with inherent ferromagnetism, large spin polarization, a high Curie temperature, and an ultralow exfoliation energy [21,22,23]. As a means of achieving polarized electronic devices, using an insulating substrate for modulation can simplify the model and avoid the influence of interlayer charge transfer on the electrical properties of the device. Researchers have successfully used CrOCl as a substrate and introduced artificial anisotropy into isotropic materials via symmetry engineering including MoS_2_/CrOCl and WSe_2_/CrOCl heterostructures [12,24]. Therefore, utilizing CrOCl as a substrate to reduce the symmetry of 2D materials is a recognized and viable approach.

In this work, we report a noteworthy enhancement effect on the in-plane anisotropy of Ta_2_NiS_5_ via vdW symmetry engineering. The experimental results of angle-resolved Raman spectroscopy demonstrated that the anisotropy enhancement occurred in the B_2g_, ^2^A_g_, and ^3^A_g_ modes of the Ta_2_NiS_5_/CrOCl heterostructure. The angle-dependent electrical transport results indicate that the anisotropy ratio of conductivity and mobility in the heterostructure increased compared with that in pristine Ta_2_NiS_5_. The Ta_2_NiS_5_/CrOCl heterostructure possesses strong anisotropic electrical properties, which can be utilized for direction-sensitive electronic devices. Our research provides a new sight for symmetry engineering in nanoelectronics.

## 2. Materials and Methods

We prepared Ta_2_NiS_5_ and CrOCl flakes (Figure S1) from bulk crystals (Onway Technology Co., Ltd., Shanghai, China) via mechanical exfoliation and constructed Ta_2_NiS_5_/CrOCl vdW heterostructures using dry transfer via transfer equipment with a 2D location adjustment platform and optical microscope. Due to the anisotropic properties of both Ta_2_NiS_5_ and CrOCl, we aligned the *a*-axis of Ta_2_NiS_5_ with the *a*-axis of CrOCl when constructing the heterostructure. We used polarization Raman spectroscopy to determine the lattice orientation of the material (Figures S2 and S3). Simultaneously, we combined the literature findings to confirm that the long-axis of Ta_2_NiS_5_ was the *a*-axis, while the long-axis of CrOCl was referred to as the *a*-axis [22,25]. In order to enhance the interlayer coupling of the heterostructure and remove the residual adhesive on the heterostructure surface, we annealed the heterostructure at 325 °C for 1 h. Six pairs of electrodes with Cr/Au (10/70 nm) were fabricated using electron-beam lithography (Raith, Pittsburgh, Germany) and PVD75 e-beam evaporation (Kurt J. Lesker, Pittsburgh, Jefferson Hills, UT, USA). For the Raman spectroscopy (WITEC, Ulm, Germany), we employed a 532 nm laser source and a 100× microscope objective. In the parallel configuration, the incident light polarization (*e_i_*) was parallel to the scattered light polarization (*e_s_*), while in the vertical configuration, *e_i_* was vertical to *e_s_*. We defined the direction as 0° when the *a*-axis of Ta_2_NiS_5_ was parallel to the incident light direction. The laser spot size was less than 500 nm. To avoid sample damage, the laser power was adjusted to less than 1 mw. The electrical characterization of the Ta_2_NiS_5_ and Ta_2_NiS_5_/CrOCl heterostructures was carried out with a probe station (Lake Shore, Westerville, OH, USA) equipped with a semiconductor analyzer system (Keithley, Cleveland, OH, USA).

## 3. Results

**Figure 1:**
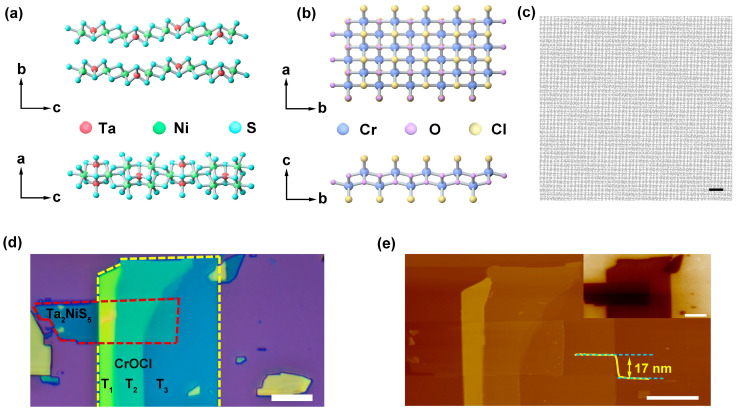
Characterizations of Ta_2_NiS_5_/CrOCl heterostructure. Schematic illustrations of the crystal lattice structure for (**a**) Ta_2_NiS_5_ and (**b**) CrOCl. (**c**) Stripe moiré pattern simulation diagram of heterointerface. The scale bar is 1 nm. (**d**) Optical image and (**e**) AFM of Ta_2_NiS_5_/CrOCl heterostructure. The thickness of Ta_2_NiS_5_ is 9.2 nm, and the thicknesses of the T_1_, T_2_, and T_3_ of CrOCl are approximately 61, 27.5, and 17 nm. The inset shows the KPFM image of Ta_2_NiS_5_/CrOCl heterostructure. The scale bars in (**d**,**e**) are both 10 μm.

We chose a ternary transition-metal chalcogenide, Ta_2_NiS_5_, and an insulator, CrOCl, as the building blocks of the interface because the compounds have similar rotational and mirror symmetries. The Ta_2_NiS_5_ and CrOCl crystal structures are illustrated in Figure 1a,b, both belonging to an orthorhombic structure [23]. When Ta_2_NiS_5_ and CrOCl are stacked to form a heterostructure, stripe moiré patterns occur at the Ta_2_NiS_5_/CrOCl interface (Figure 1c). The generation of moiré patterns further alters the electrical and optical properties of the heterostructure [26]. Unlike the moiré patterns in twisted graphene, the stripe moiré patterns originate from the lattice mismatch at the Ta_2_NiS_5_/CrOCl interface, and it may induce in-plane polarization at this interface via strain [27,28]. In a MoS_2_/CrOCl heterostructure, this stripe moiré pattern is ascribed to the lattice mismatch between MoS_2_ and CrOCl, resulting in the strain in the MoS_2_ [9,12].

Figure 1d shows the optical image of the Ta_2_NiS_5_/CrOCl heterostructure. The red-marked region indicates Ta_2_NiS_5_, and the yellow-marked region represents multiple thicknesses of CrOCl. Figure 1e corresponds to the atomic force microscope (AFM) image, which provides a higher-resolution view. It can be observed that the surface of the heterostructure is smooth. No crack or fold exists in the overlapping area, indicating the high quality of the interface. The inset is the Kelvin probe force microscopy (KPFM) image of the Ta_2_NiS_5_/CrOCl heterostructure. The KPFM image exhibits a highly uniform potential distribution in the overlapping regions, and a significant potential discrepancy can be seen.

**Figure 2:**
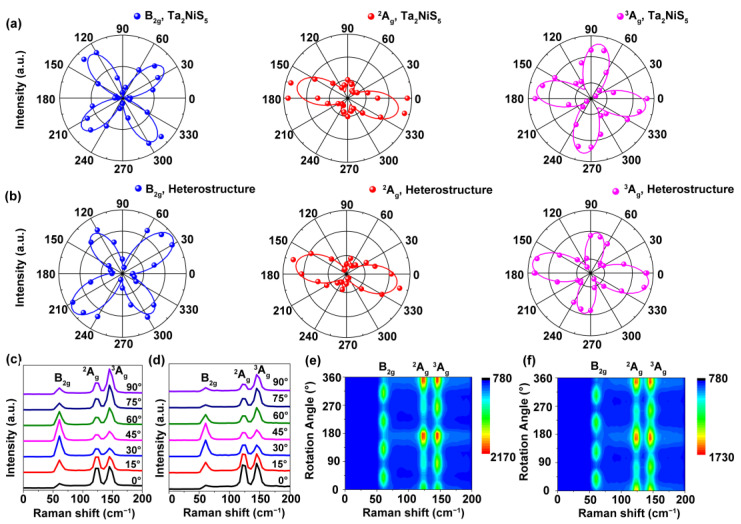
Polarized Raman spectra of Ta_2_NiS_5_/CrOCl heterostructure under parallel-polarized configuration. The polar plots of (**a**) Ta_2_NiS_5_ and (**b**) Ta_2_NiS_5_/CrOCl heterostructure for B_2g_, ^2^A_g_, and ^3^A_g_ intensities in a rotation period. Raman spectra for different polarized angles of (**c**) Ta_2_NiS_5_ and (**d**) Ta_2_NiS_5_/CrOCl heterostructure. Contour maps of angular-dependent Raman spectra of (**e**) Ta_2_NiS_5_ and (**f**) Ta_2_NiS_5_/CrOCl heterostructure.

We employed angular-resolved polarized Raman spectroscopy to investigate the symmetry of the Ta_2_NiS_5_ and Ta_2_NiS_5_/CrOCl heterostructures, aiming to uncover the influence of symmetry engineering on the in-plane polarization intensity. For Ta_2_NiS_5_, it has a B_2g_ and three A_g_ vibration modes. The force vectors correspond to a twisting motion for the B_2g_ mode and stretching motions for the ^2^A_g_ and ^3^A_g_ modes [13]. The polarization plots of each Raman mode are shown in Figure 2a,b. The B_2g_, ^2^A_g_, and ^3^A_g_ modes of pristine Ta_2_NiS_5_ and the Ta_2_NiS_5_/CrOCl heterostructure exhibit four-lobed shapes. Under the parallel polarization configuration, the B_2g_ mode intensity of pristine Ta_2_NiS_5_ had a 90° variation period, while its intensities achieved the maxima at α ≈ 40°, 130°, 220°, and 310°. The B_2g_ mode intensities achieved the maxima in the Ta_2_NiS_5_/CrOCl heterostructure at α ≈ 40° and 220° with the sub-maxima at α ≈ 30° and 310°. The anisotropy ratio of B_2g_ intensity increased from 4.6 (Ta_2_NiS_5_) to 9 (Ta_2_NiS_5_/CrOCl). Similar behavior was observed in the ^3^A_g_ mode, where the maximum intensities occurred at α ≈ 170°, and 350° for both pristine Ta_2_NiS_5_ and the heterostructure, with sub-maxima at α ≈ 80°, and 260° in the heterostructure. The anisotropy ratio of the ^3^A_g_ intensities increased from 2 (Ta_2_NiS_5_) to 3.3 (Ta_2_NiS_5_/CrOCl). Meanwhile, the ^2^A_g_ mode reached its maximum intensities at α ≈ 165° and 345° in both pristine Ta_2_NiS_5_ and the Ta_2_NiS_5_/CrOCl heterostructure, with sub-maxima at α ≈ 45° and 135°. The anisotropy ratio increased from 3.8 (Ta_2_NiS_5_) to 5.9 (Ta_2_NiS_5_/CrOCl). By comparing the Raman spectroscopy results of Ta_2_NiS_5_ and the Ta_2_NiS_5_/CrOCl heterostructure, it can be clearly observed that the asymmetry of Ta_2_NiS_5_ was enhanced by constructing the heterostructure.

As shown in Figure 2c, the B_2g_, ^2^A_g_, and ^3^A_g_ of pristine Ta_2_NiS_5_ along the *a*-axis are located at 61.6, 123.7, and 146.1 cm^−1^. The Raman frequencies of Ta_2_NiS_5_ and Ta_2_NiS_5_/CrOCl along the *a*- and *c*-axes are exhibited in Figure 2d and Table S1, wherein all the Raman frequencies of the heterostructure shift along both the *a*-axis and *c*-axis compared with those of the Ta_2_NiS_5_. Figure 2e,f shows the contour maps of the Raman intensity varying with the angle for Ta_2_NiS_5_ and the heterostructure, respectively. By comparing the Raman spectra of Ta_2_NiS_5_ and Ta_2_NiS_5_/CrOCl, it can be observed that the Raman frequency shift occurred in the heterostructure. The Raman frequency is influenced by temperature, doping, material thickness, and strain [18,29,30,31]. In our comparative experiments, the same Ta_2_NiS_5_ thickness and test temperature were used, and CrOCl was used as an insulator, eliminating the influence of doping. Therefore, we suspect that the Raman shift was mainly caused by strain. When Ta_2_NiS_5_ and CrOCl form a vdW heterostructure, the mismatch of their lattice constants leads to lattice reconstruction, reducing the symmetry of Ta_2_NiS_5_ and enhancing the polarization. Meanwhile, the lattice mismatch may also cause strain within Ta_2_NiS_5_, further affecting the symmetry of the observed Raman modes in it. The B_2g_, ^2^A_g_, and ^3^A_g_ modes represent the distortion and stretching movements of the Raman force vector. When Ta_2_NiS_5_ is strained, the Raman frequencies and symmetries of these modes change accordingly, consistent with the experimental phenomena we observed.

**Figure 3:**
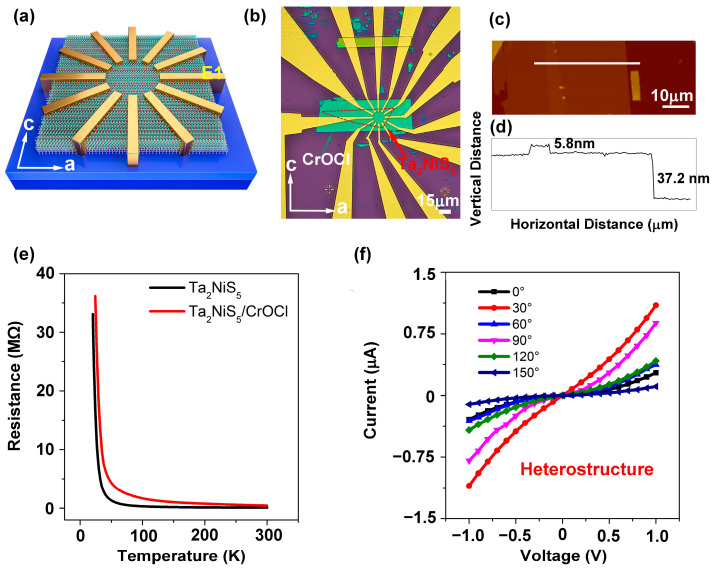
Characterization of Ta_2_NiS_5_/CrOCl device. (**a**) Schematic view of device structure. The (**b**) optical image and (**c**) AFM image of Ta_2_NiS_5_/CrOCl device. (**d**) Height map of Ta_2_NiS_5_/CrOCl device that scanned along the white area in (**c**). (**e**) Temperature-dependent resistance curves of Ta_2_NiS_5_ and Ta_2_NiS_5_/CrOCl heterostructure along *a*-axis. (**f**) I_ds_–V_ds_ curves of Ta_2_NiS_5_/CrOCl heterostructure with different angles at room temperature.

In order to further compare the enhancement effect of anisotropy on Ta_2_NiS_5_, we characterized the Ta_2_NiS_5_/CrOCl heterostructure via angle-dependent electrical transport measurements. Figure 3a shows a schematic diagram of the Ta_2_NiS_5_/CrOCl device, where we define 0° as the angle when the electrode E1 is parallel to the *a*-axis of Ta_2_NiS_5_. The side view of the device structure is shown in Figure S4. The electrode was deposited on Ta_2_NiS_5_. The channel length was 15 μm, and the angle between adjacent electrodes was 30°. Figure 3b,d displays the optical microscope image, AFM image, and height map image of the Ta_2_NiS_5_/CrOCl device. The prepared heterostructure exhibits uniform quality distribution, free of wrinkles and residue, showcasing a high-quality vdW interface. The thicknesses of Ta_2_NiS_5_ and CrOCl were 5.8 nm and 37.2 nm, respectively. The I-V curves were measured between distinct diagonal contacts at various temperatures. We tested the temperature-dependent resistance curves of Ta_2_NiS_5_ and the Ta_2_NiS_5_/CrOCl heterostructure along the *a*-axis (Figure 3e). With the increase in temperature, the resistance of the *a*-axis decreased, showing typical semiconductor characteristics, which were similar to those of the pristine Ta_2_NiS_5_. Figure 3f presents the I_ds_–V_ds_ curves of the Ta_2_NiS_5_/CrOCl heterostructure at different angles at room temperature. The currents at different angles show significant anisotropy.

**Figure 4:**
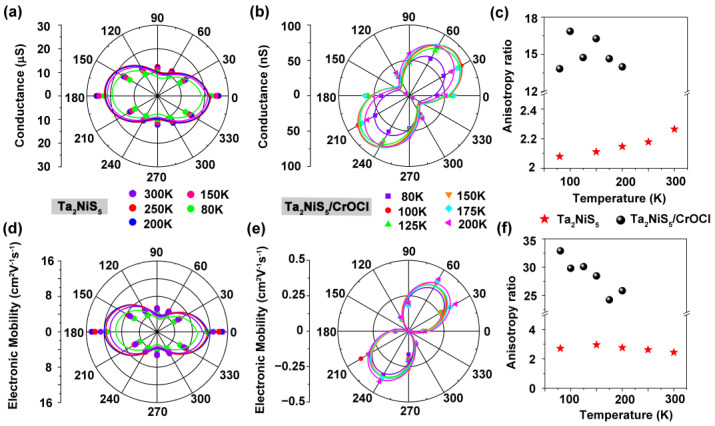
Electrical anisotropy of Ta_2_NiS_5_ and Ta_2_NiS_5_/CrOCl devices. Angle-dependent DC conductance of (**a**) Ta_2_NiS_5_ and (**b**)Ta_2_NiS_5_/CrOCl heterostructure at different temperatures. Angle-dependent electron mobility of (**d**) Ta_2_NiS_5_ and (**e**) Ta_2_NiS_5_/CrOCl heterostructure at different temperatures. (**c**) Conductivity σ and (**f**) mobility μ ratio of Ta_2_NiS_5_ and Ta_2_NiS_5_/CrOCl heterostructure at different temperatures.

To further describe the electrical anisotropy of the heterostructure, we characterized the conductivity and mobility of the Ta_2_NiS_5_ and Ta_2_NiS_5_/CrOCl devices. At a certain angle *θ*, the conductivity of anisotropic materials can be expressed as [32]:

(1) $$\sigma_{\theta }=\sigma_{a}{sin}^{2}\theta +\sigma_{c}{cos}^{2}\theta$$

where *σ_θ_* represents the conductivity of the sample in the *θ* direction, *σ_a_* and *σ_c_* denote the conductivity along the *a*- and *c*-axes, respectively. We measured the electrical conductivity at 80–300 K, as shown in Figure 4a and Table S2. The pristine Ta_2_NiS_5_ exhibited mirror symmetry, so its electrical transport properties exhibited two-fold rotational symmetry along the *a*-axis and *c*-axis [13]. The electrical conductivity reached its maximum along the *a*-axis and achieved its minimum along the *c*-axis. The anisotropy ratio of pristine Ta_2_NiS_5_ was approximately 2.1 (Figure 4c), which is similar to the reported value of 1.78–1.41 (80–300 K) in the literature [13]. Figure 4b displays a polar plot of the electrical conductivity of Ta_2_NiS_5_/CrOCl at 80–200 K, indicating clear anisotropy in conductance. The angle-dependent conductance exhibits typical two-fold symmetry. The *σ_max_* occur at *θ* = 52° and 232°, while the *σ_min_* are present at *θ* = 142° and 322°. The deviation of the polar axis might be attributed to lattice mismatch at the interface of the heterostructure, leading to a change in the periodic symmetry of the lattice. Similar phenomena have also been observed in other heterostructures [8,33]. Figure 4c shows the comparison of the anisotropy ratio (*σ_max_/σ_min_*) of the electrical conductivity of Ta_2_NiS_5_ and Ta_2_NiS_5_/CrOCl at different temperatures. The anisotropic ratio is approximately 15, which is one order higher than that of the pristine Ta_2_NiS_5_ (~2.1).

The corresponding angle-resolved transfer characteristics for Ta_2_NiS_5_/CrOCl are shown in Figure S5. The transfer curves at different temperatures and angles demonstrate considerable discrepancies. The anisotropic carrier mobility of Ta_2_NiS_5_/CrOCl was estimated according to the equation [34]:

(2) $$\mu =\left(\frac{dI_{ds}}{dV_{g}}\right)\left(\frac{L}{WC_{i}V_{ds}}\right)$$

where *L* and *W* represent the length and width of the channel. *C_i_ =* ε_0_ε*_r_/d* is the gate capacitance, ε_0_ is the vacuum dielectric constant, ε*_r_* is the relative dielectric constant of SiO_2_ (for Ta_2_NiS_5_) and CrOCl (for Ta_2_NiS_5_/CrOCl), and *d* is the thickness of SiO_2_ (for Ta_2_NiS_5_) and CrOCl (for Ta_2_NiS_5_/CrOCl). *dI_ds_/dV_g_* represents the maximum slope of the linear region in the transfer curve. The carrier mobility of pristine Ta_2_NiS_5_ exhibits a similar dependence on angles to conductivity (Figure 4d). The anisotropy ratio (*a/c*-axis) of the mobility is approximately 2.7 (Figure 4f, red). The angle-resolved field-effect carrier mobilities are shown in Figure 4e. The maximum mobility occurs at 53° (233°), whereas the minimum is at 143° (323°), giving an anisotropic mobility ratio (*μ_max_/μ_min_*) of approximately 32 at 80 K. The anisotropic ratio of mobility decreases with the increase in temperature. At 200 K, the mobilities anisotropic ratio is approximately 25 (Figure 4f, black). The anisotropic ratio of mobilities in the heterostructure is enhanced by one order of magnitude compared with that of the pristine Ta_2_NiS_5_. The experimental results of electrical transport once again demonstrate that via symmetry engineering, we successfully enhanced the in-plane anisotropy in Ta_2_NiS_5_.

Based on the above experimental results, we conclude that the in-plane anisotropy enhancement of Ta_2_NiS_5_/CrOCl might be attributed to the following reasons. The cause of enhanced anisotropy may be attributed to the reduction in lattice symmetry induced by the vdW interface constructed via symmetry engineering. Recent studies also indicate that constructing a vdW interface can reduce lattice symmetry [35,36]. By utilizing the symmetric engineering of functionalized heterointerfaces with anisotropic vdW dielectric SiP_2_, in-plane polarization was induced within the isotropic single-layer MoS_2_, resulting in anisotropic conductivity and photoluminescence [37]. Hangyel et al. studied the in-plane anisotropy of graphene induced by strong interlayer interactions with vdW epitaxially grown on MoO_3_ layers [38]. The in-plane conductivity anisotropy of graphene is 1.43. By constructing a BP/Bi_2_Se_3_ heterostructure, anisotropic optical properties were generated within the isotropic Bi_2_Se_3_, with the anisotropic ratio of polarization Raman intensity reaching up to 12 [39]. The stripe moiré patterns simulated at the Ta_2_NiS_5_/CrOCl interface also reveal that the lattice mismatch led to a decrease in the symmetry of the heterostructure. Furthermore, all the Raman frequencies shifted in the heterostructure compared with those of pristine Ta_2_NiS_5_, and the Raman frequency shift could be related to strain, excluding the effects of temperature, doping, and thickness. We speculate that the anisotropy enhancement of Ta_2_NiS_5_ may be caused by the strain induced by lattice mismatch at the vdW interface. It has been confirmed in MoS_2_/CrOCl heterostructures that lattice mismatch between MoS_2_ and CrOCl results in uniaxial strain in the MoS_2_ [12]. In addition, Ni et al. predicted that under smaller in-plane strain, anisotropy can be observed in SnSe/GeSe [40]. Thus, we infer that the enhancement of anisotropy in Ta_2_NiS_5_/CrOCl might be attributed to the vdW-interface-induced symmetry reduction and the strain.

## 4. Conclusions

In conclusion, by constructing vdW heterostructures via symmetric engineering, we demonstrated the enhancement of anisotropy in the Ta_2_NiS_5_/CrOCl heterostructure via polarized Raman spectroscopy and electrical transport measurements. Angle-resolved polarized Raman spectroscopy revealed that the polarized intensities of the B_2g_, ^2^A_g_, and ^3^A_g_ modes in the heterostructure were enhanced. The anisotropy ratios for the B_2g_, ^2^A_g_, and ^3^A_g_ modes increase from 4.6, 3.8, and 2 in the pristine Ta_2_NiS_5_ to 9, 5.9, and 3.3 in the Ta_2_NiS_5_/CrOCl heterostructure, respectively. The angle-dependent electrical transport measurements prove that the anisotropic ratio of conductivity and mobility in the heterostructure increased by one order of magnitude compared with those of the pristine Ta_2_NiS_5_. The anisotropy ratio of conductivity was enhanced from ~2.1 (Ta_2_NiS_5_) to ~15 (Ta_2_NiS_5_/CrOCl), and the anisotropy ratio of mobility was enhanced from ~2.7 (Ta_2_NiS_5_) to ~32 (Ta_2_NiS_5_/CrOCl). The reason for this anisotropic enhancement may have contributed to the lattice mismatch and strain. This study provides inspiration to study symmetry-related van der Waals heterostructures and pave the way to novel nano-electronic devices.

## Data Availability

The data presented in this study are available upon request from the corresponding authors.

## Supplementary Materials

The following supporting information can be downloaded at https://www.mdpi.com/article/10.3390/nano13233050/s1. Figure S1: Photograph of Ta_2_NiS_5_ and CrOCl; Figure S2: The angle-dependent Raman spectra of CrOCl flake in parallel configuration; Figure S3: The angle-dependent Raman spectra of CrOCl flake in perpendicular configuration; Figure S4: Schematic diagram of Ta_2_NiS_5_/CrOCl device in side view; Figure S5: Transfer characteristic curves of Ta_2_NiS_5_/CrOCl device; Table S1: Comparison of Raman frequencies of Ta_2_NiS_5_ and Ta_2_NiS_5_/CrOCl; Table S2: Comparison of the anisotropy ratios of Ta_2_NiS_5_ and Ta_2_NiS_5_/CrOCl heterostructure.
