# Supplementary material for: Symmetry-Engineering-Induced In-Plane Polarization Enhancement in Ta2NiS5/CrOCl van der Waals Heterostructure
Source: Nanomaterials (Basel). 2023 Nov 29;13(23):3050. doi: 10.3390/nano13233050 (PMC10708406; doi:10.3390/nano13233050)
Supplement: Supplementary file 1 [file nanomaterials-13-03050-s001.zip › nanomaterials-2737443-supplementary.pdf]

Supplementary Materials

Symmetry-Engineering-Induced In-Plane Polarization Enhancement in Ta_2NiS_5/CrOCl van der Waals Heterostructure

Yue Su,[1,2] Peng Chen,[3] Xiangrui Xu,[1] Yufeng Zhang,[1] Weiwei Cai,[1] Gang Peng,[2] Xueao Zhang,[1*] Chuyun Deng [2*]

1 College of Physical Science and Technology, Xiamen University, Xiamen 361005, China

2 College of Science, National University of Defense Technology, Changsha 410073, China

3.Songshan Lake Materials Laboratory, Dongguan 523808, China

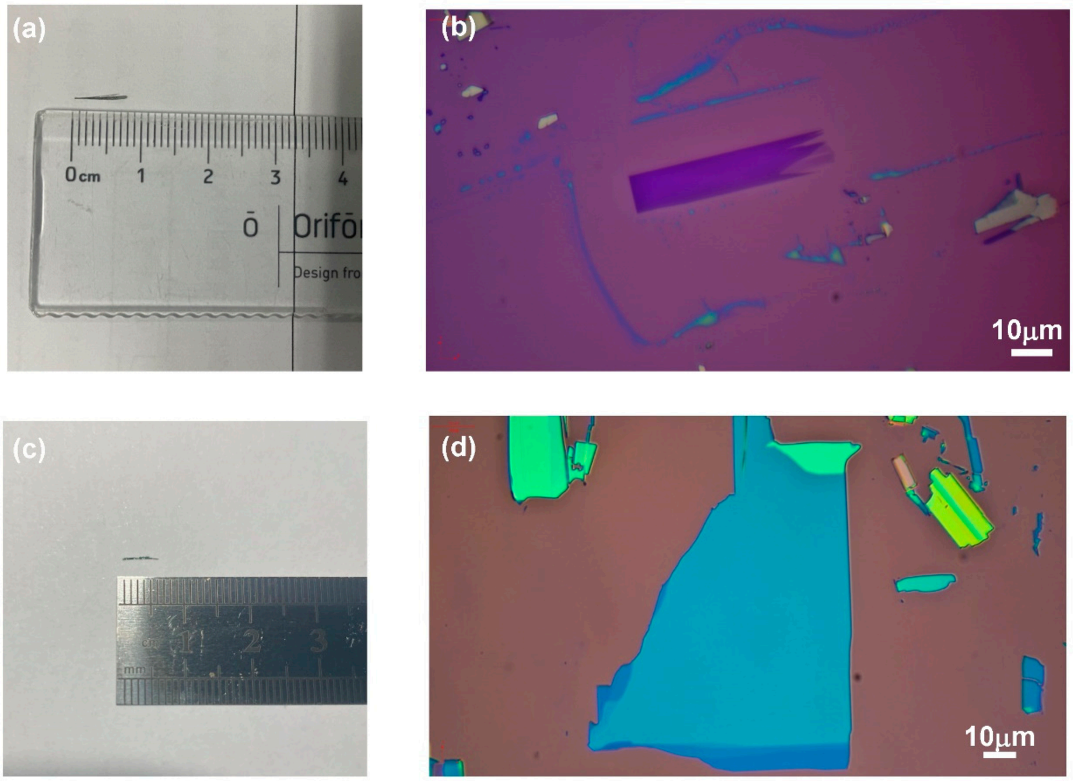

Figure S1. Photograph of bulk (a) Ta_2NiS_5 and (c) CrOCl crystal. Optical microscopy image of few-layer (b) Ta_2NiS_5 and (d) CrOCl flake. The scale bar is 10μm.

Table S1. Comparison of Raman frequencies of Ta_2NiS_5 and Ta_2NiS_5/CrOCl.

	$B_{2g,a\text{-}axis}$	$^2A_{g,a\text{-}axis}$	$^3A_{g,a\text{-}axis}$	$B_{2g,c\text{-}axis}$	$^2A_{g,c\text{-}axis}$	$^3A_{g,c\text{-}axis}$
Ta_2NiS_5	61.6cm^{-1}	123.7cm^{-1}	146.1cm^{-1}	60.7cm^{-1}	123.7cm^{-1}	146cm^{-1}
Ta_2NiS_5/CrOCl	61.2cm^{-1}	124.1cm^{-1}	146.5cm^{-1}	60.9cm^{-1}	123.9cm^{-1}	146.4cm^{-1}
Frequency shift	-0.4cm^{-1}	0.4cm^{-1}	0.4cm^{-1}	0.2cm^{-1}	0.2cm^{-1}	0.4cm^{-1}

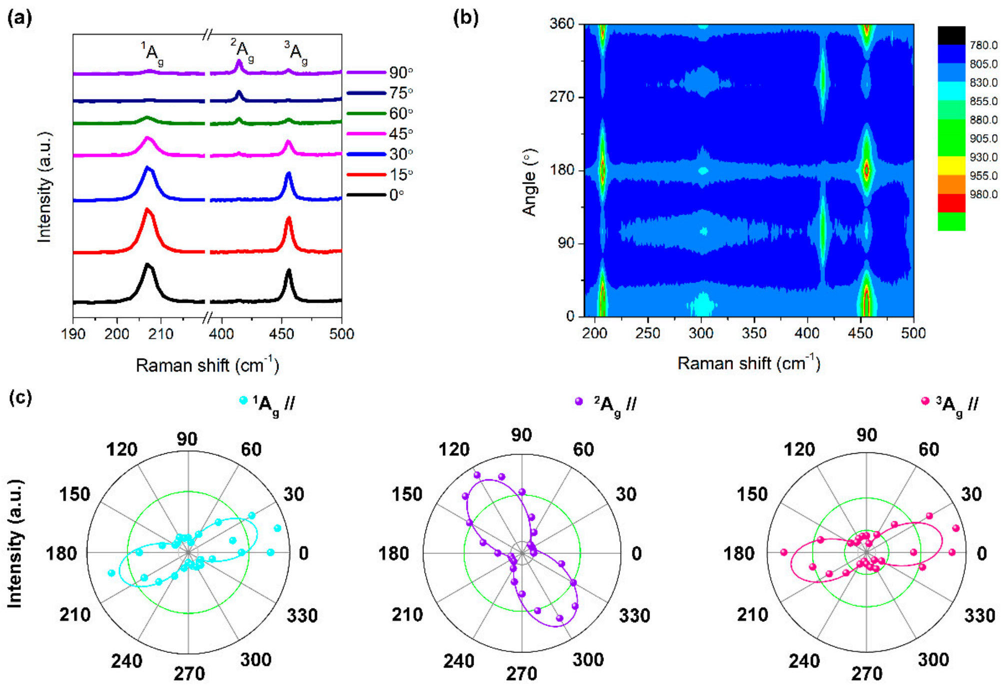

Figure S2. In parallel configurations, (a) polarized Raman spectra, (b) corresponding contour map and (c) polar plots of CrOCl flake.

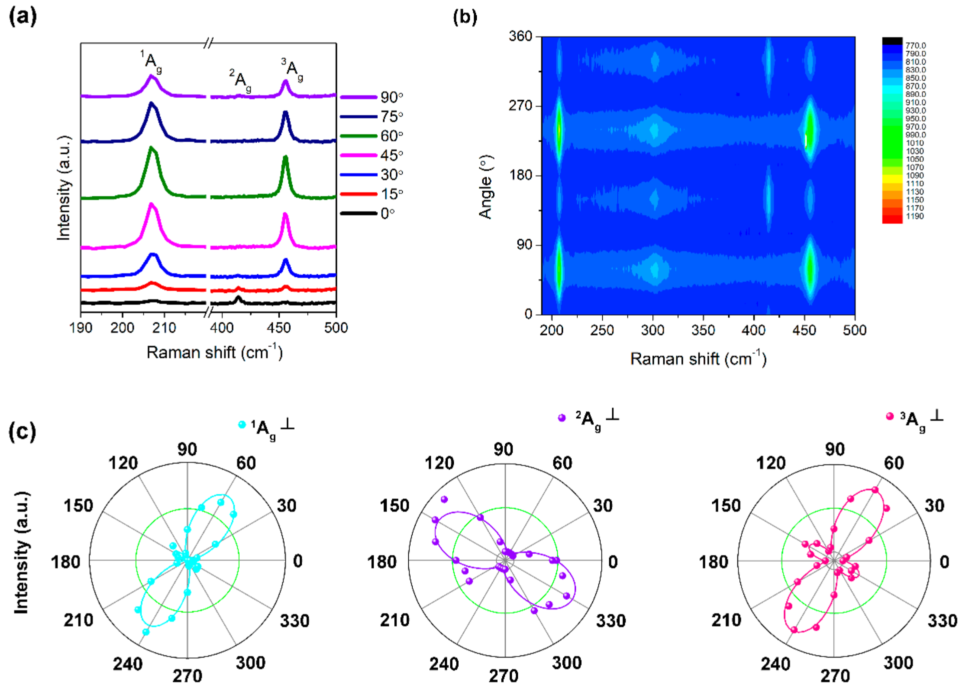

Figure S3. In perpendicular configurations, (a) polarized Raman spectra, (b) corresponding contour map and (c) polar plots of CrOCl flake.

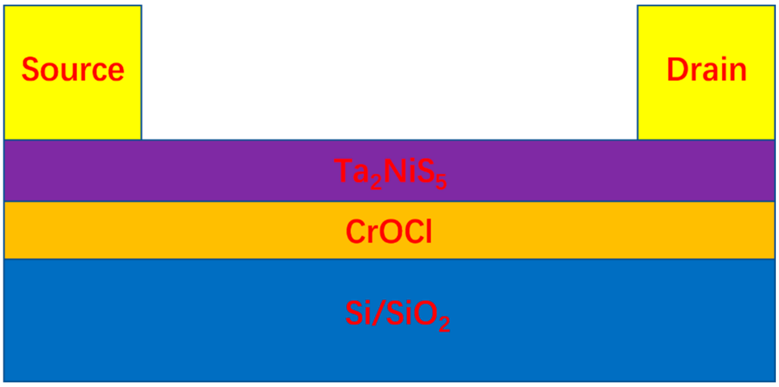

Figure S4. Schematic diagram of Ta_2NiS_5/CrOCl device in side view.

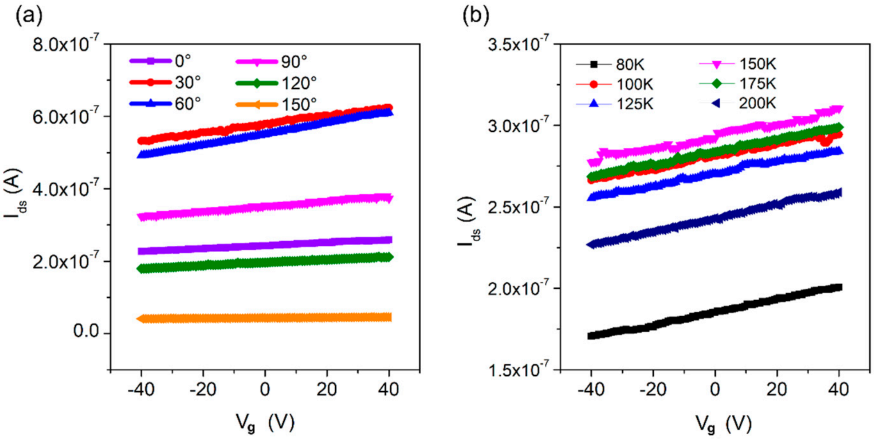

Figure S5. (a) Transfer characteristic curves of Ta_2NiS_5/CrOCl along *a*-axis at different temperatures. (b) Transfer characteristic curves of Ta_2NiS_5/CrOCl along different angles at 200K.

Table S2. Comparison of the anisotropy ratio of Ta_2NiS_5 and Ta_2NiS_5/CrOCl heterostructure.

Materials	σ_a/σ_c	μ_a/μ_c	Reference
Ta_2NiS_5	1.78	/	[1]
Ta_2NiS_5	2.1	2.7	This work
Ta_2NiS_5/CrOCl	15	32	This work

References:

1. Li, L.; Gong, P.; Wang, W.; Deng, B.; Pi, L.; Yu, J.; Zhou, X.; Shi, X.; Li, H.; Zhai, T. Strong In-Plane Anisotropies of Optical and Electrical Response in Layered Dimetal Chalcogenide. *ACS Nano* **2017**, *11*, 10264-10272, doi:10.1021/acsnano.7b04860.
